# Sex-Specific Vulnerabilities in Lung Adenocarcinoma Among Non-Smoking Women: A Conceptual Review of Multisystem Pathways and Preventive Implications

**DOI:** 10.3390/cancers18020266

**Published:** 2026-01-15

**Authors:** Ren-Jen Hwang, Hsiu-Chin Hsu, Yueh-O Chuang

**Affiliations:** 1 Department of Nursing, Chang Gung University of Science and Technology, Taoyuan 333324, Taiwan; 2 Department of Nursing, Linkou Chang Gung Memorial Hospital, Taoyuan 333, Taiwan; j22092@gmail.com; 3 Department of Graduate Institute of Gerontology and Health Care Management, Chang Gung University of Science and Technology, Gueishan 333324, Taiwan; hchsu@mail.cgust.edu.tw; 4 Department of Internal Medicine, Chang Gung Memorial Hospital, Taoyuan 333, Taiwan

**Keywords:** lung adenocarcinoma, non-smoking women, multisystem vulnerability, EGFR, tumor microenvironment, conceptual review

## Abstract

**Background:** Lung adenocarcinoma in non-smoking women represents a distinct clinical entity that cannot be fully explained by traditional exposure-centered carcinogenic models. Although ambient air pollution is a recognized risk factor, sex-specific vulnerability suggests the involvement of additional biological modulators shaping inflammatory, immune, and proliferative responses. **Main body:** In this conceptual review, we integrate epidemiological, experimental, and mechanistic evidence to propose a multisystem framework of lung carcinogenesis in non-smoking women. We delineate a central carcinogenic spine encompassing lung epithelial injury, chronic inflammation, growth factor signaling activation—particularly epidermal growth factor receptor (EGFR) pathways—and tumor microenvironment remodeling. Within this framework, three interacting domains function as biological modulators that amplify carcinogenic processes: chemosensory–neural–immune modulation, hormonal–endocrine signaling including estrogen–EGFR crosstalk, and psychosocial stress–hypothalamic–pituitary–adrenal (HPA) axis dysregulation. These domains converge through feedback mechanisms that reinforce systemic dysregulation and tumor-promoting microenvironments. **Implications:** This integrative model provides a biologically grounded perspective on female-specific vulnerability to lung adenocarcinoma and informs precision prevention, risk stratification, and ESG-informed public health strategies beyond conventional exposure reduction.

## Simple Summary

Lung adenocarcinoma in women who have never smoked is increasingly recognized as a distinct disease entity. Although air pollution is an established risk factor, differences in susceptibility between women and men suggest that additional biological mechanisms are involved. In this conceptual review, we propose a multisystem framework in which sensory processing, hormonal regulation, and stress-related neuroendocrine pathways interact to amplify inflammation, immune dysregulation, and tumor-promoting signaling in the lung. By integrating evidence across these domains, we highlight how female-specific biological and psychosocial factors may contribute to lung cancer development beyond exposure alone. This perspective provides a foundation for mechanism-informed prevention strategies and public health approaches tailored to non-smoking women.

## 1. Introduction

Lung adenocarcinoma among women who have never smoked has emerged as a distinct and increasingly recognized clinical and epidemiological entity. Despite substantial declines in tobacco use worldwide, the incidence of lung cancer in never-smokers—particularly among women—remains persistently high, accounting for a significant proportion of global lung cancer burden [1,2,3]. This pattern challenges traditional carcinogenic paradigms that prioritize direct genotoxic exposure to tobacco smoke as the dominant explanatory mechanism.

Large-scale epidemiological analyses consistently demonstrate that women exhibit higher susceptibility to lung cancer at comparable or even lower levels of ambient air pollution exposure than men [4,5]. Fine particulate matter (PM_2.5_) and traffic-related pollutants have been implicated not only in lung cancer initiation but also in tumor promotion and progression, with evidence suggesting sex-specific differences in biological response [6,7]. However, prevailing risk models often treat sex as a demographic covariate rather than as a biologically embedded determinant of vulnerability, thereby limiting their capacity to explain why non-smoking women are disproportionately affected.

Recent advances in molecular and experimental oncology further indicate that air pollution acts through mechanisms extending beyond direct DNA damage. Experimental studies demonstrate that particulate matter can promote clonal expansion of oncogenic mutations, activate inflammatory and immune-modulatory pathways, and interact with growth factor signaling cascades such as epidermal growth factor receptor (EGFR), which is frequently mutated in lung adenocarcinoma among never-smoking women [6,7,8,9]. These findings underscore the need for conceptual frameworks that integrate exposure, host biology, and systemic regulation.

Importantly, emerging evidence suggests that environmental exposures should not be conceptualized solely as chemical insults but also as sensory and psychosocial stressors that engage multiple physiological systems. Airborne pollutants interact with the respiratory epithelium, olfactory and trigeminal pathways, neuroendocrine signaling, and immune surveillance mechanisms, all of which exhibit sex-specific modulation [10,11,12,13]. Women generally demonstrate heightened chemosensory sensitivity, distinct hormonal regulation, and differential stress responsivity, raising the possibility that lung adenocarcinoma in non-smoking women reflects a form of multisystem vulnerability rather than a single-exposure disease [12,14,15,16].

The purpose of this article is to reframe lung adenocarcinoma among non-smoking women through a conceptual review lens. Rather than conducting a systematic synthesis of all available studies, we integrate representative epidemiological, experimental, and clinical evidence to propose a multisystem framework linking chemosensory processing, endocrine modulation, and psychosocial stress pathways. These representative studies were selected based on their relevance to biological plausibility, documented sex-specific patterns, and their capacity to inform cross-domain integration, rather than to provide exhaustive coverage. Such reframing has implications not only for mechanistic understanding but also for prevention strategies, public health governance, and environmentally informed cancer control policies.

## 2. Multisystem Mechanistic Pathways Underlying Female-Specific Vulnerability

### 2.1. From Exposure-Centered Models to Multisystem Vulnerability

Conventional models of lung carcinogenesis have largely emphasized dose–response relationships between inhaled toxicants and direct cellular DNA damage. While this framework has been instrumental in establishing causal links between tobacco smoke, occupational exposures, and lung cancer, it is less capable of explaining sex-specific patterns observed among never-smoking populations [2,3,17]. In particular, these models do not adequately account for why women may experience heightened lung cancer risk at lower or comparable levels of ambient air pollution exposure.

A multisystem vulnerability perspective offers an alternative conceptual orientation. Rather than attributing disease susceptibility to isolated exposures, this framework emphasizes dynamic interactions among multiple physiological systems that shape how environmental stressors are perceived, processed, and biologically internalized. In the context of lung adenocarcinoma among non-smoking women, vulnerability may be amplified through coordinated engagement of chemosensory pathways, endocrine regulation, and psychosocial stress responses. This integrative framing is proposed as a conceptual synthesis by the present review, rather than as a mechanism established by any single experimental study. The relative relevance of each evidence domain to non-smoking female LUAD is summarized in Table 1.

### 2.2. Chemosensory and Olfactory Pathways as Exposure Amplifiers

Women generally exhibit superior olfactory sensitivity and lower odor detection thresholds compared with men, a difference that has been consistently demonstrated across psychophysical, neuroanatomical, and meta-analytic studies [18,19,20]. Sex differences in olfactory function are evident across the lifespan and appear to be partially modulated by reproductive hormones, particularly estrogen, which influences both peripheral olfactory receptor activity and central olfactory processing [18,19]. These differences suggest that equivalent airborne pollutant concentrations may elicit qualitatively distinct sensory and neural responses across sexes.

Epidemiological studies further indicate that exposure to ambient air pollutants is associated with measurable alterations in olfactory function among women, implicating the olfactory system as a biologically relevant interface between inhaled exposures and downstream physiological responses [13,18,19]. Neuroanatomically, olfactory and trigeminal pathways provide direct connections to limbic and hypothalamic regions involved in emotion, stress regulation, and autonomic control [10,12]. Experimental and neuroimaging studies demonstrate that olfactory stimulation—particularly when odors are perceived as unpleasant or threatening—can modulate stress-related neural circuits and influence neuroendocrine signaling [2,21].

From a mechanistic standpoint, repeated or chronic chemosensory engagement with environmental pollutants may therefore function as an exposure amplifier, transforming low-level environmental contact into sustained neuroendocrine and immune perturbations. This proposed amplification mechanism represents an integrative interpretation advanced by the present review, rather than a causal pathway definitively established by existing studies.

### 2.3. Hormonal Modulation and Endocrine–Immune Crosstalk

Sex hormones play a central role in modulating lung tissue homeostasis, cellular proliferation, and immune surveillance. Estrogen receptors are expressed in both normal lung epithelium and lung adenocarcinoma cells, and estrogen signaling has been shown to interact with growth factor pathways such as epidermal growth factor receptor (EGFR) signaling [2,22]. EGFR mutations are disproportionately prevalent in lung adenocarcinoma among never-smoking women, particularly in East Asian populations, underscoring the relevance of hormone–growth factor interactions in female-specific carcinogenesis [7,8,23,24].

Environmental pollutants further complicate this regulatory landscape by acting as endocrine-disrupting agents. Components of ambient particulate matter and other environmental estrogens have been shown to interfere with estrogen receptor and G protein-coupled estrogen receptor (GPER) signaling, potentially enhancing proliferative cues or attenuating immune-mediated tumor suppression [25,26]. Longitudinal cohort studies also suggest associations between air pollution exposure and alterations in circulating reproductive hormone levels, particularly during hormonally dynamic life stages such as the menopausal transition [27,28].

Within a multisystem framework, hormonal modulation is not viewed as an isolated molecular mechanism but as a nodal process linking environmental exposures to immune and inflammatory responses. This perspective helps explain why similar pollutant exposures may yield divergent carcinogenic trajectories across sexes and life stages, even in the absence of direct genotoxic injury [4,16,29].

### 2.4. Psychosocial Stress, HPA Axis Dysregulation, and Immune Consequences

Psychosocial stress represents a third, often underappreciated, domain of vulnerability. Women disproportionately experience chronic stressors related to caregiving responsibilities, socioeconomic inequities, and cumulative emotional labor, factors that have been associated with long-term adverse health outcomes [14,30,31,32]. Chronic activation of the hypothalamic–pituitary–adrenal (HPA) axis can result in sustained cortisol dysregulation, immune suppression, and pro-inflammatory states, all of which may facilitate tumor initiation and progression [14,33,34].

Environmental exposures themselves may function as psychosocial stressors. Persistent exposure to malodorous or irritating air pollutants has been linked to heightened stress perception, sleep disturbance, and somatic symptom reporting [35,36]. Experimental studies further demonstrate that olfactory stimulation can modulate stress-response neural circuits, providing a mechanistic bridge between sensory exposure and neuroendocrine dysregulation [21]. Air pollution has also been associated with altered diurnal cortisol patterns, supporting a role for environmental stressors in HPA axis perturbation [21,37].

Chronic stress may additionally contribute to immune aging and reduced tumor surveillance through mechanisms such as telomere shortening and altered T-cell function [14,38]. Within a multisystem vulnerability framework, these processes represent pathways through which social and environmental stressors become biologically embedded over time.

### 2.5. Integrating the Domains: Toward a Unified Mechanistic Perspective

Taken together, chemosensory amplification, hormonal modulation, and psychosocial stress form an interconnected network rather than discrete pathways. Sensory processing can potentiate stress responses; hormonal regulation can shape immune consequences of stress; and psychosocial factors can modulate both sensory perception and endocrine signaling. The present review proposes that lung adenocarcinoma among non-smoking women emerges from cumulative multisystem imbalance rather than single-agent causation, a conceptual orientation that aligns mechanistic evidence across domains without attributing this integration to any single study.

## 3. Evidence Landscape Across Three Interconnected Domains

In a conceptual review, evidence serves not as a comprehensive inventory of studies but as a foundation for theory building and integrative interpretation. Accordingly, this section synthesizes representative epidemiological, experimental, and clinical studies selected for their relevance to sex-specific vulnerability, biological plausibility, and cross-domain coherence, rather than for exhaustive coverage. Rather than emphasizing study counts or methodological hierarchies, we focus on converging lines of evidence that contribute mechanistic insight, demonstrate sex-specific patterns, and support cross-domain integration [4,6,39]. The evidentiary role of each domain is categorized as direct, partial, or indirect, as summarized in Table 1.

### 3.1. Chemosensory Sensitivity and Sensory–Neural Interfaces with Environmental Exposure

Large-scale epidemiological studies consistently associate ambient air pollution—particularly fine particulate matter (PM_2.5_) and traffic-related pollutants—with increased lung cancer risk among never-smokers [4,5]. Importantly, several pooled cohort analyses and regional studies report stronger associations in women than in men, suggesting that sex-specific susceptibility contributes to observed disparities beyond differences in exposure magnitude [16].

While most epidemiological models interpret these findings through dose-based toxicological frameworks, experimental and neurobiological evidence highlights the sensory dimension of exposure as a potential modifier of risk. Olfactory and trigeminal pathways provide a direct interface between inhaled pollutants and central nervous system regions involved in affective processing and stress regulation [10,16,20,40]. Population-based studies further demonstrate associations between ambient air pollution and impaired olfactory function among women, supporting the biological relevance of chemosensory pathways in environmental exposure processing [13].

Experimental and neuroimaging studies indicate that unpleasant or irritating odors can activate limbic circuitry, increase pain perception, and modulate autonomic and stress-related neural responses [11,13,21,41]. These findings provide mechanistic plausibility for the notion that repeated low-level chemosensory stimulation may translate environmental exposure into sustained neural and neuroendocrine engagement. The interpretation of chemosensory sensitivity as an exposure amplifier represents an integrative perspective advanced by the present review, rather than a causal claim established by any single study. As summarized in Table 1, evidence related to chemosensory and olfactory pathways is considered partially relevant to lung adenocarcinoma in non-smoking women, insofar as it informs sensory–neural interfaces and exposure amplification rather than direct carcinogenic endpoints

### 3.2. Hormonal Regulation, Endocrine Disruption, and EGFR-Related Pathways

Hormonal regulation constitutes a second domain in which evidence supports female-specific vulnerability to lung adenocarcinoma. Estrogen receptors are widely expressed in lung tissue, and estrogen signaling has been implicated in cellular proliferation, inflammatory modulation, and interaction with oncogenic pathways in non-small cell lung cancer [22,41,42,43]. Clinical and molecular studies further indicate that estrogen can interact with EGFR signaling, a pathway disproportionately activated in lung adenocarcinoma among never-smoking women [8,16,24]. Within this domain, evidence ranges from partial mechanistic support (e.g., endocrine disruption and immune modulation) to direct relevance where estrogen–EGFR interactions intersect with established lung adenocarcinoma pathways (Table 1).

Environmental exposures may influence these processes through endocrine-disrupting effects. Longitudinal cohort studies report associations between ambient air pollution exposure and alterations in circulating reproductive hormone levels among women, including during the menopausal transition [26]. Experimental studies also demonstrate that environmental estrogens and particulate matter components can activate estrogen receptor-mediated and GPER-mediated signaling pathways, potentially enhancing proliferative signaling or attenuating immune surveillance [26,44].

Experimental oncology studies provide further support for this interactional model. Air pollution has been shown to promote lung adenocarcinoma progression and clonal expansion through inflammatory and receptor-mediated mechanisms, even in the absence of direct mutagenesis [7,28,45]. Collectively, these findings support a role for endocrine–immune crosstalk in shaping female-specific carcinogenic trajectories. The integration of hormonal modulation with environmental exposure is proposed here as a conceptual framework, rather than as a single experimentally validated pathway.

### 3.3. Psychosocial Stress, HPA Axis Dysregulation, and Biological Embedding

Psychosocial stress represents a third line of evidence relevant to multisystem vulnerability. Women disproportionately experience chronic stressors related to caregiving, unpaid domestic labor, and socioeconomic inequities, factors that have been associated with adverse physical health outcomes and increased mortality risk [4,16,30]. Chronic stress has been shown to dysregulate the hypothalamic–pituitary–adrenal (HPA) axis, leading to sustained alterations in cortisol secretion, immune suppression, and pro-inflammatory signaling [14,33,34].

Environmental exposures may further exacerbate these processes. Studies demonstrate that persistent exposure to malodorous air pollution is associated with heightened stress perception, sleep disturbance, and somatic symptom reporting [35,36]. Experimental evidence indicates that olfactory stimulation can directly modulate stress-response neural circuits, providing a mechanistic bridge between sensory exposure and HPA axis activation [21]. Air pollution exposure has also been linked to altered diurnal cortisol patterns in population-based studies, supporting a role for environmental stressors in neuroendocrine dysregulation [37].

Chronic stress and neuroendocrine imbalance may contribute to immune aging and reduced tumor surveillance through mechanisms such as telomere shortening and altered T-cell function [14,14,46]. These findings support the concept of biological embedding, whereby repeated psychosocial and environmental stressors become internalized as long-term physiological alterations relevant to cancer susceptibility. Consistent with the conceptual scope of this review, psychosocial stress-related evidence is treated as an indirect contributor to multisystem vulnerability, rather than as direct evidence of lung adenocarcinoma causation (Table 1).

### 3.4. Sex-Related Contrasts in Multisystem Vulnerability Pathways

Although the present review focuses on lung adenocarcinoma among non-smoking women, contrasting hypothesized vulnerability pathways between women and men provides important contextual clarification. Existing epidemiological evidence consistently indicates that women exhibit higher lung adenocarcinoma incidence than men at comparable or lower levels of ambient air pollution exposure, suggesting that sex-related biological factors may modify susceptibility beyond exposure magnitude alone [2,3].

Within the chemosensory domain, multiple psychophysical and neurobiological studies report greater olfactory sensitivity and lower detection thresholds in women than in men [18,19]. While enhanced sensory acuity does not imply pathological vulnerability per se, it may plausibly increase the frequency or intensity of neural engagement with airborne pollutants. At present, evidence directly linking sex-related differences in olfactory mucosal structure, epithelial permeability, or ciliary function to carcinogenic susceptibility remains limited. Available human data primarily support sex differences in olfactory sensitivity and central neural processing rather than well-characterized structural divergence, indicating that this pathway should be regarded as indirect and hypothesis-generating rather than causal.

Hormonal modulation represents a more directly supported domain of sex-related divergence. Estrogen receptor expression in lung tissue, interactions between estrogen signaling and EGFR-related pathways, and the higher prevalence of EGFR mutations among never-smoking women together suggest a biologically plausible axis through which environmental exposures may yield sex-differentiated oncogenic trajectories [24,42]. In contrast, corresponding endocrine–oncogenic interactions in men appear less pronounced or are mediated through distinct hormonal milieus, although direct comparative studies remain scarce.

Psychosocial stress and HPA axis regulation further contribute to sex-related contrast. Population-level data indicate that women, on average, experience greater cumulative psychosocial stress related to caregiving roles, socioeconomic inequities, and unpaid labor, factors associated with sustained neuroendocrine and immune alterations [31,33,34]. While chronic stress is not specific to women, sex differences in stress perception, coping, and hormonal responsivity may influence biological embedding processes relevant to cancer susceptibility. Evidence in this domain is largely indirect and context-dependent.

Importantly, these contrasts should not be interpreted as deterministic or exclusive to women. Rather, the present review proposes that the convergence of modest sex-related differences across sensory, endocrine, and psychosocial domains may collectively shape differential vulnerability profiles. This integrative perspective underscores the need for future studies explicitly designed to compare women and men across these interacting systems, rather than inferring sex differences from single-domain evidence alone. A conceptual summary of these contrasts is provided in Table 2.

### 3.5. Cross-Dimensional Integration and Prevention Implications

Across chemosensory, hormonal, and psychosocial domains, evidence converges on a common theme: vulnerability arises from interaction rather than isolation. Sensory processing can potentiate stress responses; hormonal regulation can modulate immune consequences of stress; and psychosocial factors can shape both sensory perception and endocrine signaling [6,39]. Epidemiological observations of sex disparities in lung adenocarcinoma risk among never-smokers are consistent with this interactional model, even though no single study has empirically integrated all domains simultaneously [5,17].

By emphasizing feedback and cross-system interaction rather than linear causation, the evidence landscape supports a shift away from single-factor explanations toward a systemic understanding of disease susceptibility. This synthesis provides the conceptual bridge to cross-domain integration, which is developed in the following section. Table 3 Summary of Representative Evidence Supporting Multisystem Vulnerability in Non-Smoking Women.

## 4. Cross-Domain Integration: Toward a Multisystem Model of Female Vulnerability

**Figure 1:**
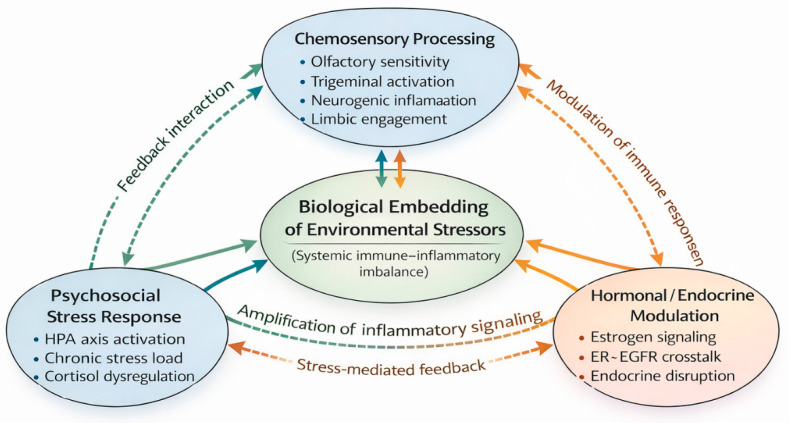
Conceptual network model illustrating multisystem vulnerability in non-smoking women. Figure 1 presents a conceptual, theory-generating framework illustrating how chemosensory processing, hormonal modulation, and psychosocial stress responses may interact to shape multisystem vulnerability in non-smoking women. Each domain represents a functional biological module supported by converging epidemiological, experimental, and clinical evidence, rather than a direct causal pathway to lung adenocarcinoma. Solid arrows denote biologically plausible interactions supported by existing evidence, whereas dashed arrows indicate hypothesized or indirect feedback processes. The central node (“biological embedding/systemic imbalance”) reflects the cumulative internalization of environmental and psychosocial exposures, emphasizing interaction and amplification rather than linear causation.

The evidence reviewed across chemosensory, hormonal, and psychosocial domains suggests that lung adenocarcinoma among non-smoking women cannot be adequately explained by linear, exposure-centered models alone. Instead, these domains appear to interact through reciprocal feedback loops that shape how environmental stressors are perceived, processed, and biologically embedded over time. Figure 1 illustrates this proposed multisystem model, emphasizing interaction rather than isolated causation.

At the sensory–neural interface, heightened chemosensory sensitivity in women may amplify environmental exposures by increasing neural and affective engagement with airborne pollutants. Olfactory and trigeminal inputs directly access limbic and hypothalamic circuits involved in stress regulation and autonomic control, providing a pathway through which repeated low-level exposures can elicit sustained neuroendocrine responses [10,12,21]. These sensory-driven stress responses may, in turn, modulate hormonal signaling and immune function, thereby influencing downstream carcinogenic processes.

Hormonal modulation represents a second node of integration. Estrogen signaling interacts with growth factor pathways such as EGFR, which are frequently activated in lung adenocarcinoma among never-smoking women [8,16,24]. Endocrine-disrupting pollutants may further perturb this balance by altering estrogen receptor-mediated and GPER-mediated signaling [26,44]. Within the proposed framework, hormonal regulation is not an isolated molecular mechanism but a dynamic mediator that shapes tissue responsiveness to inflammatory and proliferative cues generated by both sensory and stress-related inputs.

Psychosocial stress provides a third axis of integration, linking environmental perception to systemic biological consequences. Chronic activation of the hypothalamic–pituitary–adrenal (HPA) axis has been associated with immune suppression, altered inflammatory profiles, and accelerated biological aging [34,46]. Sensory exposure to environmental pollutants—particularly when perceived as unpleasant or uncontrollable—may function as a stressor in its own right, reinforcing HPA axis dysregulation and sustaining feedback loops between neural, endocrine, and immune systems [21,35].

Crucially, these domains are likely to operate in a bidirectional and mutually reinforcing manner. Sensory amplification may heighten stress responsivity; hormonal states may modulate sensory perception and immune resilience; and psychosocial stress may alter both endocrines signaling and neural processing of environmental stimuli. The present review proposes that lung adenocarcinoma among non-smoking women emerges from cumulative multisystem imbalance, rather than from the direct effect of any single exposure or pathway. This integrative interpretation reflects a conceptual synthesis grounded in converging evidence, rather than a mechanistic model established by a single experimental paradigm.

By explicitly incorporating feedback and cross-domain interaction, the multisystem vulnerability model provides a coherent explanation for observed sex disparities in lung adenocarcinoma risk and molecular characteristics. It also establishes a mechanistic rationale for prevention strategies that extend beyond exposure reduction alone, thereby linking conceptual integration with actionable research and policy considerations.

From a translational perspective, the multisystem framework proposed here also lends itself to experimentally testable models. For example, in vitro lung epithelial cell systems could be exposed to defined concentrations of PM_2.5_ or traffic-related pollutants under estrogen-modulated conditions, enabling direct examination of how hormonal signaling influences pollutant-induced inflammatory responses, EGFR pathway activation, or clonal expansion dynamics. Such models would allow controlled dissection of endocrine–environmental interactions relevant to lung adenocarcinoma in non-smoking women.

In parallel, stress-mimicking experimental paradigms could be incorporated by modulating glucocorticoid signaling or HPA-axis-related pathways in epithelial or immune–epithelial co-culture systems. These approaches would permit investigation of how chronic stress-associated neuroendocrine signals interact with sensory and hormonal pathways to influence immune surveillance and tumor-promoting microenvironments. Importantly, these examples are proposed as hypothesis-generating platforms rather than validated disease models, and they illustrate how the conceptual framework outlined in this review can inform future mechanistic and translational research.

## 5. Prevention, ESG, and Public Health Implications

**Figure 2:**
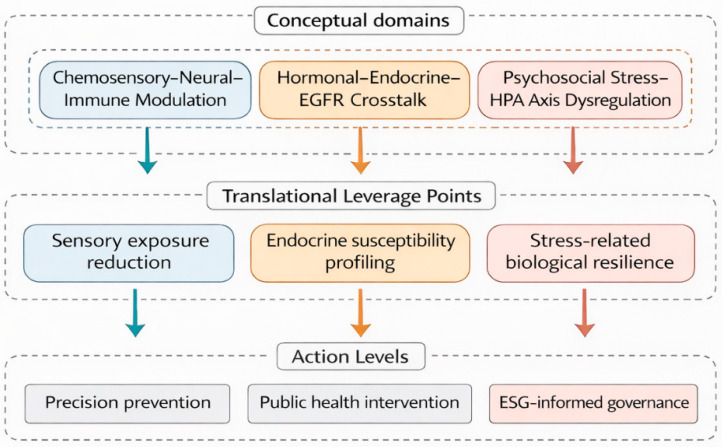
Translational and preventive implications of multisystem vulnerability in non-smoking women. Mechanistic domains identified in Figure 1—chemosensory–neural–immune modulation, hormonal–endocrine–EGFR crosstalk, and psychosocial stress–HPA axis dysregulation—are translated into downstream biological processes, clinical risk contexts, and psychosocial determinants relevant to lung adenocarcinoma among non-smoking women. These interconnected pathways highlight actionable leverage points across molecular, individual, and population levels, informing integrated strategies for precision prevention, public health intervention, and ESG-informed governance aimed at reducing female-specific vulnerability.

The multisystem vulnerability framework outlined above has important implications for cancer prevention, public health strategy, and governance. If lung adenocarcinoma risk among non-smoking women arises from the interaction of chemosensory amplification, hormonal modulation, and psychosocial stress, then prevention efforts must move beyond single-exposure reduction toward approaches that address cumulative and context-dependent risk. Figure 2 summarizes a prevention-oriented framework informed by this multisystem perspective, spanning environmental control, individual-level resilience, and structural governance.

### 5.1. Mechanism-Informed Primary Prevention

At the environmental level, primary prevention remains anchored in reducing population exposure to ambient air pollution, particularly PM_2.5_ and traffic-related pollutants, which have been consistently linked to lung cancer risk even at low concentrations [4,5]. Evidence indicating that air pollutants can promote tumor initiation and progression through inflammatory and receptor-mediated pathways underscores the importance of stringent air quality standards and continuous monitoring [6,7,45]. From a multisystem perspective, exposure reduction may also mitigate sensory and stress-related pathways by decreasing the frequency and intensity of aversive environmental stimuli.

Mechanism-informed prevention further suggests that attention to indoor air quality and consumer chemical exposures is warranted. Household products, occupational settings, and urban microenvironments may contribute to persistent low-level chemosensory stimulation and endocrine disruption, particularly among women with caregiving roles and prolonged indoor exposure. While direct causal links to lung adenocarcinoma require further study, existing evidence supports precautionary approaches that limit cumulative chemical burden.

### 5.2. Secondary Prevention and Risk Stratification

The recognition of sex-specific vulnerability pathways has implications for secondary prevention and early detection. Never-smoking women are often underrepresented in traditional lung cancer screening paradigms, which prioritize smoking history as the primary risk criterion [15,17]. A multisystem framework supports the development of refined risk stratification models that incorporate environmental exposure profiles, hormonal factors, and stress-related indicators alongside molecular markers such as EGFR mutation status [9,23].

Emerging evidence that chemosensory disturbances may precede or accompany lung cancer diagnosis suggests potential avenues for symptom-informed surveillance, although this remains an exploratory area [47]. The present review proposes that integrating sensory, endocrine, and psychosocial indicators into risk assessment could enhance early detection strategies, a hypothesis that warrants prospective evaluation rather than immediate clinical adoption.

### 5.3. Psychosocial and Gender-Sensitive Interventions

Psychosocial stress mitigation represents an additional, often overlooked, component of cancer prevention. Chronic caregiving stress, unpaid labor, and gendered social roles have been associated with sustained neuroendocrine and immune dysregulation among women [30,31,32]. Accordingly, interventions that reduce chronic stress—by strengthening social support systems, reforming workplace policies, promoting healthy lifestyles, and expanding access to mental health resources—may play an important indirect role in enhancing cancer-related biological resilience [14,33,34,38,48,49].

From a multisystem perspective, such interventions are not merely adjunctive but may influence core biological pathways implicated in vulnerability. Reducing stress-related HPA axis activation could attenuate immune suppression and inflammatory signaling, thereby modulating downstream carcinogenic processes. These considerations highlight the importance of gender-sensitive public health strategies, particularly in populations where women face disproportionate environmental and social burdens.

### 5.4. ESG, Environmental Justice, and Governance

The multisystem vulnerability framework also aligns with environmental, social, and governance (ESG) principles. Environmental injustice disproportionately exposes women—especially those in low-income or urban settings—to air pollution and chemical hazards, compounding biological and psychosocial vulnerability [48,50]. Integrating cancer prevention into ESG-informed policy frameworks emphasizes accountability for environmental emissions, transparency in chemical regulation, and equitable distribution of health-protective resources.

At the governance level, incorporating mechanistic insights into regulatory decision-making may strengthen the rationale for precautionary policies, even in the absence of definitive causal proof for every pathway. The present review argues that multisystem vulnerability provides a biologically grounded justification for preventive governance, linking environmental regulation, occupational safety, and social policy to cancer control objectives.

### 5.5. Implications for Research and Policy Integration

Finally, the framework outlined here underscores the need for interdisciplinary research designs capable of capturing cumulative and interactive effects. Longitudinal studies integrating environmental exposure assessment, hormonal profiling, stress biomarkers, and molecular tumor characteristics are essential for testing the hypotheses generated by this conceptual model. Such approaches may inform both individualized prevention strategies and population-level policy interventions.

In summary, prevention strategies informed by multisystem vulnerability extend beyond exposure reduction to encompass gender-sensitive, mechanism-aware, and governance-oriented approaches. This perspective bridges mechanistic understanding with actionable public health and policy considerations, setting the stage for a balanced discussion of limitations and future directions.

## 6. Limitations and Future Directions

Several limitations of the present conceptual review should be acknowledged. First, by design, this article does not aim to provide a systematic or exhaustive synthesis of all available studies. Instead, it integrates representative epidemiological, experimental, and clinical evidence to support a multisystem vulnerability framework. As a result, the proposed interactions among chemosensory processing, hormonal modulation, and psychosocial stress should be interpreted as hypothesis-generating rather than as definitive causal pathways.

Second, much of the existing evidence supporting individual domains derives from studies conducted in isolation, often with differing populations, exposure metrics, and outcome definitions. Epidemiological studies linking air pollution to lung cancer risk typically emphasize exposure intensity and duration, whereas neurobiological and psychosocial studies focus on sensory processing and stress-related outcomes [5,13,14,21]. The lack of integrative study designs limits the ability to empirically test cross-domain interactions within a single cohort.

Third, sex-specific mechanisms discussed here may be influenced by additional biological and social modifiers that were not explicitly addressed. Genetic susceptibility, life-course exposures, reproductive history, and cultural context may further shape vulnerability among non-smoking women [16,28,29]. While these factors are compatible with the multisystem framework, they require dedicated investigation to clarify their relative contributions and interactions.

Despite these limitations, the conceptual model outlined in this review highlights several priorities for future research. Longitudinal studies that integrate environmental exposure assessment, chemosensory function, hormonal profiling, stress biomarkers, and molecular tumor characteristics are needed to test the proposed pathways. Experimental models that explicitly examine sex differences and hormonal status in response to environmental stressors may further elucidate mechanistic links. Finally, interdisciplinary approaches combining oncology, neuroscience, endocrinology, and social science are essential for translating multisystem vulnerability concepts into actionable prevention strategies.

## 7. Conclusions

Lung adenocarcinoma among non-smoking women represents a growing public health challenge that cannot be fully explained by traditional exposure-centered carcinogenic models. Accumulating evidence indicates that women experience heightened susceptibility to environmental risk through mechanisms that extend beyond direct genotoxic injury, involving chemosensory processing, hormonal modulation, and psychosocial stress regulation.

In this conceptual review, we propose a multisystem vulnerability framework that integrates these domains into a coherent explanatory model. Rather than attributing disease risk to any single pathway, this framework emphasizes interaction, feedback, and biological embedding as key features shaping female-specific carcinogenic trajectories. Importantly, this synthesis reflects an interpretive integration of existing evidence, not a claim that any single study has empirically established all proposed links.

By reframing lung adenocarcinoma in non-smoking women as a multisystem condition, this perspective offers new avenues for research, prevention, and policy. Mechanism-informed prevention strategies that incorporate environmental control, gender-sensitive risk stratification, psychosocial support, and governance considerations may better address the complex and cumulative nature of vulnerability. Ultimately, advancing cancer prevention for non-smoking women will require approaches that recognize the intertwined biological and social contexts in which environmental exposures occur.

## Figures and Tables

**Table 1:** Relevance of Evidence Domains to Lung Adenocarcinoma in Non-Smoking Women.

Evidence Domain	Type of Evidence	Representative Focus	Relevance to Non-Smoking Female LUAD
Ambient air pollution and lung carcinogenesis	Epidemiological, molecular, experimental oncology	PM_2.5_ exposure, chronic inflammation, EGFR-related signaling, tumor promotion	Direct
Chemosensory and olfactory pathways	Population-based studies, neurobiological and sensory research	Sex differences in olfactory sensitivity, sensory–neural processing of airborne pollutants	Partial
Hormonal and endocrine modulation	Clinical, molecular, and translational studies	Estrogen receptor signaling, endocrine–EGFR crosstalk, immune modulation	Partial to Direct
Psychosocial stress and HPA axis dysregulation	Population-level, psychoneuroendocrine, mechanistic studies	Chronic stress exposure, cortisol dysregulation, immune suppression	Indirect

Note: Relevance categories are used to clarify the evidentiary role of each domain within this conceptual review. Domains classified as “Partial” or “Indirect” are interpreted as modulatory contributors to multisystem vulnerability, rather than as independent or sufficient causal pathways for lung adenocarcinoma.

**Table 2:** Conceptual Comparison of Hypothesized Multisystem Vulnerability Pathways between Women and Men.

Domain	Women	Men	Representative Evidence
Chemosensory sensitivity	Generally higher olfactory sensitivity and lower detection thresholds; stronger affective and neural responses to odors reported	Lower average olfactory sensitivity; less pronounced neural engagement with odor stimuli	Sex differences in olfactory sensitivity documented in psychophysical and meta-analytic studies [18,19]
Airway/olfactory structure	Limited data on sex differences in mucosal thickness, ciliary function, or epithelial permeability; evidence remains indirect	Comparable paucity of direct comparative data	Reviews on airway epithelium and mucociliary biology note insufficient sex-stratified data [2]
Hormonal–endocrine modulation	Estrogen receptor expression in lung tissue; interaction with EGFR signaling; higher EGFR mutation prevalence among never-smoking women	Androgen-dominant milieu; weaker or distinct hormone–growth factor interactions	Estrogen–EGFR crosstalk and female-predominant EGFR mutations reported in LUAD [42]
Endocrine disruption by pollutants	Greater susceptibility during hormonally dynamic life stages (e.g., menopausal transition)	Effects reported but less consistently linked to lung oncogenic pathways	Air pollution and endocrine disruption across female life stages [8]
Psychosocial stress exposure	Higher cumulative caregiving and social stress burden on average	Different stress profiles; often occupationally patterned	Gender differences in chronic stress exposure and caregiving burden [31,34]
HPA axis and immune consequences	Evidence of sex-specific stress responsivity, cortisol reactivity, and immune modulation	Comparable mechanisms with potentially different thresholds or patterns	Sex differences in HPA axis reactivity and psychoneuroimmunology [33]
Overall vulnerability profile	Convergence of sensory, hormonal, and stress-related modifiers	Fewer converging modifiers identified in never-smokers	Conceptual synthesis of multisystem vulnerability (this review)

**Table 3:** Summary of Representative Evidence Supporting Multisystem Vulnerability in Non-Smoking Women.

Domain	Representative Evidence Type	Key Epidemiological/Mechanistic Insight	Relevance to Female-Specific Vulnerability
Chemosensory sensitivity	Population-based cohorts; neurobiological studies	Women show higher olfactory sensitivity and stronger neural responses to airborne pollutants	Sensory amplification may enhance stress and inflammatory signaling under chronic exposure
Hormonal/endocrine modulation	Cohort studies; molecular and experimental models	Estrogen signaling interacts with EGFR and pollutant-related pathways	Endocrine milieu may amplify tumor-promoting responses in non-smoking women
Psychosocial stress/HPA axis	Longitudinal and stress-biology studies	Chronic stress and cortisol dysregulation impair immune surveillance	Gendered stress exposure may contribute to cumulative biological vulnerability

Table 3 provides a conceptual synthesis of representative epidemiological and mechanistic evidence. It is not intended as a comprehensive systematic evidence table, but as an interpretive summary supporting the multisystem framework proposed in this conceptual review.

## Data Availability

The original contributions presented in this study are included in the article. Further inquiries can be directed to the corresponding author.
